# Enhanced Activity of Hierarchical Nanostructural Birnessite-MnO_2_-Based Materials Deposited onto Nickel Foam for Efficient Supercapacitor Electrodes

**DOI:** 10.3390/nano10101933

**Published:** 2020-09-27

**Authors:** Shang-Chao Hung, Yi-Rong Chou, Cheng-Di Dong, Kuang-Chung Tsai, Wein-Duo Yang

**Affiliations:** 1 Fuzhou Polytechnic, Fuzhou 350108, China; schung99@gmail.com; 2 Intelligent Technology Research Centre, Fuzhou 350108, China; 3 Department of Chemical and Materials Engineering, National Kaohsiung University of Science and Technology, Kaohsiung 80778, Taiwan; mygirl850629@gmail.com; 4 Department of Marine Environmental Engineering, National Kaohsiung University of Science and Technology, Kaohsiung 81157, Taiwan; cddong@nkust.edu.tw; 5 Department of Safety, Health and Environmental Engineering, National Kaohsiung University of Science and Technology, Kaohsiung 82445, Taiwan; tsaikc@nkust.edu.tw

**Keywords:** hierarchical, birnessite-MnO_2_, specific capacitance, electrochemical performance

## Abstract

Hierarchical porous birnessite-MnO_2_-based nanostructure composite materials were prepared on a nickel foam substrate by a successive ionic layer adsorption and reaction method (SILAR). Following composition with reduced graphene oxide (rGO) and multiwall carbon nanotubes (MWCNTs), the as-obtained MnO_2_, MnO_2_/rGO and MnO_2_/rGO-MWCNT materials exhibited pore size distributions of 2–8 nm, 5–15 nm and 2–75 nm, respectively. For the MnO_2_/rGO-MWCNT material in particular, the addition of MWCNT and rGO enhanced the superb distribution of micropores, mesopores and macropores and greatly improved the electrochemical performance. The as-obtained MnO_2_/rGO-MWCNT/NF electrode showed a specific capacitance that reached as high as 416 F·g^−1^ at 1 A·g^−1^ in 1 M Na_2_SO_4_ aqueous electrolyte and also an excellent rate capability and high cycling stability, with a capacitance retention of 85.6% after 10,000 cycles. Electrochemical impedance spectroscopy (EIS) analyses showed a low resistance charge transfer resistance for the as-prepared MnO_2_/rGO-MWCNT/NF nanostructures. Therefore, MnO_2_/rGO-MWCNT/NF composites were successfully synthesized and displayed enhanced electrochemical performance as potential electrode materials for supercapacitors.

## 1. Introduction

Recently, supercapacitors (SCs) have attracted widespread attention and research due to their advantages, such as high specific capacitance, high power density, good cycle life, and low maintenance cost [1,2,3]; however, the storage energy density for such devices is not as good as that found for lithium-ion batteries, which, thus, limits applications. In addition, the pore size, specific surface area, surface functional groups, and conductivity for an electrode active material can also affect the specific capacitance of a supercapacitor [4,5]. Therefore, improving nanostructure electrode materials with high specific capacitance to increase their specific capacitance and energy density is a focus of research for energy storage devices [6,7,8].

Manganese dioxide (MnO_2_) has the advantages of low cost, high environmental safety and high theoretical capacitance (1380 F·g^−1^) in aqueous electrolytes and is considered to be one of the most promising capacitor materials [9,10]. The common crystal structures of MnO_2_ are α, β, γ, δ and λ type crystal phases. The size of the pore structure in each crystal is different. At present, various crystal type structures have been widely studied. Among such structures, birnessite-MnO_2_ (δ-MnO_2_) is widely used as a positive active material for supercapacitors [11,12]. Devaraj et al. [13] pointed out that δ-MnO_2_ has a MnO_6_ octahedron structure due to the crystal sharing double bonds with the neighboring crystal lattice; the width of δ-MnO_2_ is large enough to accommodate the insertion or removal of cations with a large ionic radius. Wolff et al. [14] published δ-MnO_2_ as a two-dimensional layered structure, which can hold water and cations between a large number of sheet materials. Brousse et al. [15] studied the electrochemical properties of MnO_2_ with different crystal structures. The results show that the layered α-MnO_2_ and δ-MnO_2_ can rapidly diffuse potassium ions to improve the capacitance performance. Ghodbane et al. [16] pointed out that δ-MnO_2_ is more suitable for the insertion and removal of ions, and shows ionic conductivity and increased electrical properties.

According to the principles of ion adsorption-desorption and intercalation, the electrochemical capacitance behavior mainly occurs on the surface layer of the manganese dioxide electrode material. Therefore, increasing the specific surface area can effectively improve the utilization of the active material, resulting in a higher specific capacitance. However, the application of MnO_2_ in electrodes still shows some shortcomings, such as low conductivity, which severely limits the application value [17]. Fortunately, this weakness can be improved by introducing high-conductivity carbon materials into electrode materials [18]. In recent years, reduced graphene oxide (rGO) has been shown to exhibit excellent electronic, capacitance, and mechanical properties, chemical stability, and a high specific surface area. It is often compounded with metal oxides and used as an electrode material for supercapacitors [19,20]. It is worth mentioning that the aggregation and restacking of particles during the electrode preparation process for graphene results in a significant decrease in electrochemical performance. Therefore, by combining graphene and multiwall carbon nanotubes (MWCNTs), one can obtain graphene/MWCNT composite materials, which exhibit a unique structure that has attracted the attention of researchers. This composite material containing graphene and carbon nanotubes shows good electrical conductivity and excellent chemical stability, which can effectively promote the electron transfer properties of the electrolyte in the electrode material [21,22]. Particularly, MnO_2_ composited with MWCNT and graphene can effectively improve the material aggregation in the electrode and improve the performance of the supercapacitor.

Successive ionic layer adsorption and reaction (SILAR) is a chemical solution deposition technology. Using this method can coat the required thickness of film on the substrate, which is a simple process [23,24]. Jana et al. [25] prepared MnO_2_ cathode materials on stainless steel substrates by hydrothermal method or SILAR technology, respectively. Graphene particles were deposited on nanocarbon cloth as anodes to fabricate rGO-MnO_2_ asymmetric supercapacitors. It indicated that the rGO-MnO_2_ material synthesized by hydrothermal method had serious agglomeration, which reduced the conductivity and surface area of the electrode. On the contrary, the cathode material made by SILAR technology had a high surface area and a higher diffusion rate of electrolyte. Moreover, the SILAR process not only prevents the aggregation of graphene, but also eliminates the necessity of nonconductive organic binders and carbon black in the process to reduce the manufacturing cost of supercapacitor electrodes. The SILAR method is used in rGO-MnO_2_ composite films, which can manufacture a lightweight and ultrasmall super capacitor. Jadhav et al. [26], who also studied SILAR technology, used stainless steel as the substrate and immersion in manganese ion solutions by the SILAR technology. It was shown that the prepared MnO_2_ film was an amorphous particle, which exhibited an electrochemical oxidation-reduction reaction during charge and discharge. SILAR technology can uniformly adhere Mn_3_O_4_/GO to the substrate, and at 5 mV·S^−1^, the specific capacitance is 344 F·g^−1^. However, to the best of our knowledge, utilizing the SILAR process to fabricate δ-MnO_2_-based electrode materials has been very scarce and is worthy of study.

In this study, an environmentally friendly and low-cost process was developed to produce high-performance supercapacitor electrode materials. SILAR was used to prepare MnO_2_-based composite electrode materials on nickel foam (NF), such as MnO_2_/NF, MnO_2_/rGO/NF and MnO_2_/rGO-MWCNT/NF composite cathode materials to improve the agglomeration for such electrode materials. The specific surface area, pore size and distribution for the electrode materials were studied, and mixtures of rGO and MWCNT with different weight ratios were studied to investigate the electrochemical properties of the composite electrodes.

## 2. Materials and Methods

### 2.1. Materials

Analytical-grade chemicals were used as received, without any further purification. Nickel foam (98% purity, Fluka, Tokyo, Japan), graphene oxide sheet (99% purity, Goal Bio, Hsichu, Taiwan), multiwalled carbon nanotube (MWCNT) (98% purity, Showa, Tokyo, Japan), sodium sulfate (≥98% purity, Honeywell, Hannover, Germany), safranin (99% purity, Alfa Aesar, Karlsruhe, Germany), potassium permanganate (98% purity, Showa, Tokyo, Japan), and manganese sulfate (98% purity, Showa, Tokyo, Japan) were used for the preparation of the MnO_2_-based/NF electrode. Sodium sulfate (≥98% purity, Honeywell, Hannover, Germany) was used as the electrolyte.

### 2.2. Preparation of MnO_2_-Based/NF Electrode

A graphene sheet weighing 0.25 g was weighed and added into 50 mL DI water. After ultrasonic mixing for 1 h, ammonia water was used to adjust the pH at approximately 11. Then, the as-prepared GO suspension was put in an autoclave and kept at 180 °C for 12 h. After cooling, the suspension solution was turned into rGO. Finally, it was dried in a freeze dryer for 24 h. The properties of the as-obtained materials were characterized (Supplementary Materials, Figure S1).

The nickel foam (1 cm × 1 cm) was washed with acetone, ultrasonic vibration, and deionized water, and then dried. Then, 0.5 mg mL^−1^ of safranin was mixed in DI water with an initial mixture of rGO and MWCNT at a concentration of 0.6 mg·mL^−1^ in DI water. The weight ratio for rGO and MWCNT was 1:1. The mixture was then subjected to an ultrasonic vibrator for 30 min to form a uniform suspension rGO-MWCNT ink.

The cleaned NF was immersed in reduced graphene oxide (rGO), MWCNT or dispersed rGO-MWCNT ink, and then rinsed with deionized water for 40 s to obtain rGO, MWCNT or rGO-MWCNT adhered to the NF substrate to prepare rGO/NF, MWCNT/NF or rGO-MWCNT/NF, respectively.

**Figure 1:**
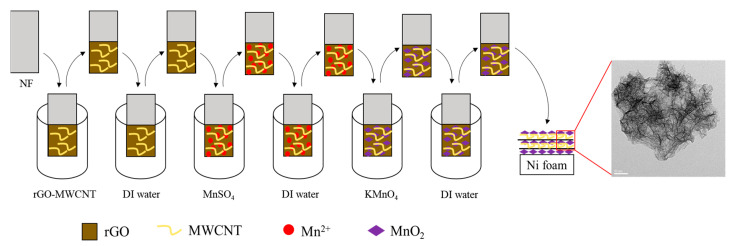
Schematic for the preparation of MnO_2_/rGO-MWCNT/NF electrode via the successive ion layer adsorption and reaction method.

SILAR technology was used to coat the film onto the substrate. NF, rGO/NF, MWCNT/NF or rGO-MWCNT/NF was respectively placed into Mn^2+^ solution (0.01 M MnSO_4_ solution) and MnO_4_^-^ solution (0.01 M KMnO_4_ solution), and then rinsed and air-dried. Mn^2+^ was oxidized and deposited layer by layer, which then underwent a redox reaction with MnO_4_^−^. Through the SILAR process, MnO_2_, MnO_2_/rGO, MnO_2_/rGO-MWCNT was attached to the Ni substrate, respectively. This procedure was repeated five times. Finally, the as-deposited electrode was annealed at 200 °C for 1 h. Figure 1 shows a schematic for the preparation of MnO_2_/rGO-MWCNT/NF material via the successive ion layer adsorption and reaction method.

### 2.3. Characterization

The crystal phases for the samples were examined using X-ray diffraction (XRD, Bruker, D8 ADVANCE, Karlsruhe, Germany) with Cu K_α_ radiation (λ = 1.5406 Å). Furthermore, transmission electron microscopy (TEM) (JEOL, TEM-3010, Tokyo, Japan) with an acceleration voltage of 80 kV was utilized to examine the microstructures for the as-prepared MnO_2_-based electrodes. The Brunauer-Emmett-Teller (BET) surface area, Barrett-Joyner-Halenda (BJH) mesopore area, t-plot micropore area, and N_2_ adsorption-desorption isotherms were measured with a Micrometrics ASAP 2020 instrument.

### 2.4. Electrochemical Characterization of the As-Obtained MnO_2_-Based/NF Electrodes

All the electrochemical properties were investigated using a conventional three-electrode electrochemical cell equipped with the as-prepared MnO_2_-based/NF electrode (1.0 cm × 1.0 cm) as the working electrode, a platinum plate (1.0 cm × 1.0 cm) as the counter electrode, and an Ag/AgCl as the reference electrode, and a 1.0 M Na_2_SO_4_ aqueous solution as the electrolyte.

Cyclic voltammetry (CV) and galvanostatic charging-discharging (GCD) tests were performed using a CHI 760D electrochemical workstation (CH Instruments, Inc., Austin, TX, USA).

The specific capacitance can be evaluated by the CV test using the following Formula (1):

(1) $$C=\frac{Q}{V\times m\times \Delta U}$$

where *Q* is the area of the CV curve, *V* is the scan rate (V·s^−1^), *m* is the mass of the electrode active material (g), and ∆*U* is the voltage range (V).

The specific capacitance was obtained according to the discharge curve of the GCD test by using the Formula (2):

(2) $$C_{m}=\frac{i\times \Delta t}{\Delta V\times m}$$

where *i* (A) is the discharge current, Δ*t* (s) is the discharge time, Δ*V*(V) is the discharging potential difference, and *m* (g) is the mass of the loaded active material (MnO_2_).

Electrochemical impedance spectra (EIS) were measured at open-circuit voltage, with a bias of 10 mV for frequencies ranging from 100 kHz to 0.01 Hz, to analyze the electron transport properties.

## 3. Results and Discussion

### 3.1. Characterization of the MnO_2_-Based Electrode Materials

The deposition of thin films was done by the following the steps to grow nucleation sites, and then ions reacted to produce the as-deposited material on the substrate, in the SILAR process. In this case, Mn^2+^ from manganese sulfate was adsorbed on the nickel foam and reacted with MnO_4_^−^ from potassium permanganate to produce MnO_2_.

In the preliminary experiment, MnO_2_ prepared under different manganese ion concentrations was characterized by XRD, BET, CV and GCD analyses. The results showed that δ-MnO_2_ crystals were obtained at a lower concentration of MnSO_4_ (0.01 M), and γ-MnO_2_ crystals were prepared at concentrations of MnSO_4_ higher than 0.05 M. Because δ-MnO_2_ crystal possessed a larger BET specific surface area and better pore properties, it exhibited better electrochemical characteristics (Supplementary Materials, Figure S2), therefore, preparation of δ-MnO_2_ was conducted on 0.01 M MnSO_4_ in this study.

**Figure 2:**
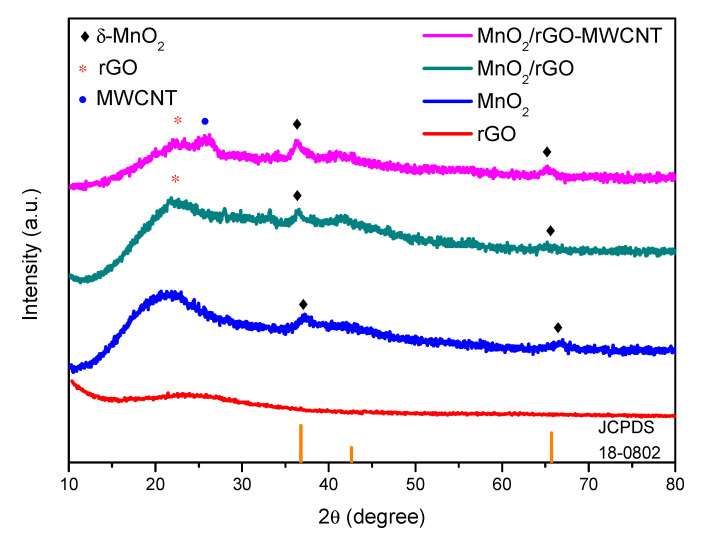
The X-ray diffraction (XRD) analysis for the MnO_2_-based electrode materials prepared by the successive ionic layer adsorption and reaction (SILAR) process.

Figure 2 shows the XRD analysis for the MnO_2_-based electrode materials prepared by the SILAR process. The results show that a weak diffraction peak can be mainly attributed to the prepared sample, which indicates low crystallinity. Nevertheless, it can still be clearly found that the diffraction peaks at 2θ values of 37.2° and 66.8° were due to the (111) and (020) crystal planes of the birnessite-type MnO_2_ (JCPDS 18-0802, Joint Committee on Powder Diffraction Standards (JCPDs), Newtown Square, PA, USA) [27] and that the peak width of the peak intensity was not strong. It is revealed that the MnO_2_ material prepared by the SILAR method was not highly crystalline and that the crystallinity was not perfect. A clear diffraction peak was observed at a 2θ value of 22° for the as-deposited MnO_2_/rGO material, showing the presence of rGO [28]; the small peak on the right of 22°, indicates that a small amount of unreacted graphite carbon existed in the GO. According to XRD analysis for the as-deposited MnO_2_/rGO-MWCNT/NF electrode material, the diffraction peak observed at a 2θ value of approximately 22° was characteristic of rGO; the peak observed at a 2θ value of approximately 25° was the characteristic peak for the MWCNTs. By using Bragg’s Law, nλ = 2d sinθ, the interplanar spacing for rGO and MWCNT was calculated to be 0.40 nm and 0.36 nm, respectively.

Birnessite MnO_2_ has a two-dimensional (2D) layered structure, consisting of MnO_6_ octahedrons shared with edges. The spacing of this layered structure is approximately 7 Å and it is located in the middle layer area, which is considered to be favorable for the transport of metal ions or water [29]. The specific capacitance of the relatively open layered structure of δ-MnO_2_ is much higher than that of β-MnO_2_ and γ-MnO_2_ [30]. A detailed comparison of the XRD diffraction peaks for the three different electrode materials composed of MnO_2_ (such as the two diffraction peaks at 2θ values of 37.2° and 66.8°) shows that the diffraction peak due to the MnO_2_/rGO-MWCNT/NF electrode material broadened and shifted to a low diffraction angle; it can be presumed that the addition of MWCNT widened the crystal planes in the prepared electrode structure (such as the (111) and (020) crystal planes).

**Figure 3:**
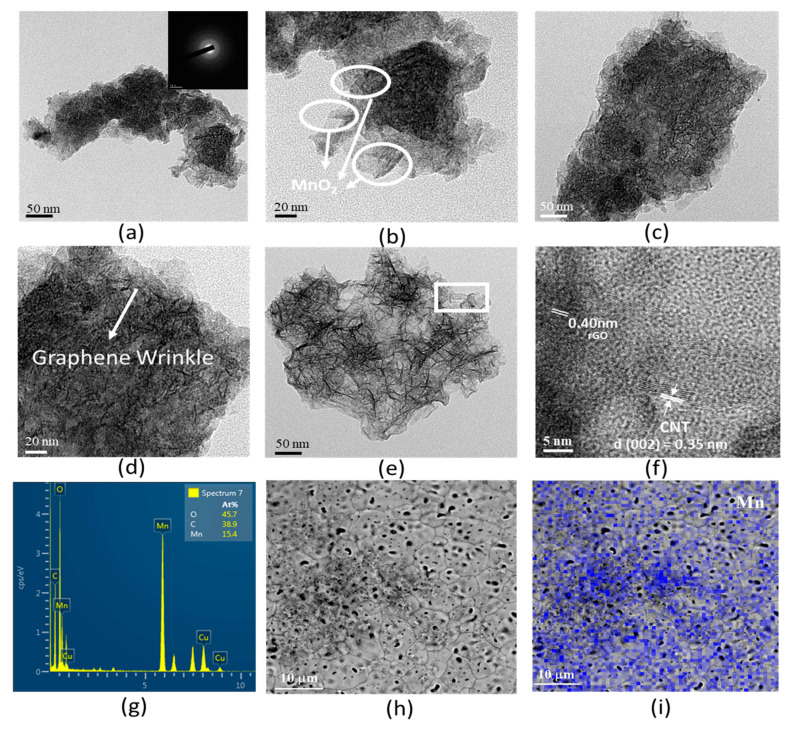
The TEM analysis for the as-obtained MnO_2_ material (**a**,**b**), MnO_2_/rGO (**c**,**d**) and MnO_2_/rGO-MWCNT material (**e**,**f**), EDS of MnO_2_/rGO-MWCNT/NF material (**g**), SEM and mapping of MnO_2_/rGO-MWCNT/NF material (**h**,**i**), respectively.

TEM was used to study the morphologies of the MnO_2_-based materials. Figure 3 shows the TEM analysis for the as-obtained MnO_2_, MnO_2_/rGO and MnO_2_/rGO-MWCNT materials. As shown in Figure 3a,b, it can be clearly seen that the prepared MnO_2_ layered structure was loosely arranged. In addition, the selected region electron diffraction image shows an indistinct electron diffraction ring (upper right corner of Figure 3a), indicating that the prepared MnO_2_ had poor crystallinity.

TEM analysis of the MnO_2_/rGO/NF nanocomposite electrode is shown in Figure 3c,d. Wrinklelike features for graphene can be observed. Because the MnO_2_/rGO/NF was prepared by the SILAR process, the graphene could restack, resulting in excessive surface agglomeration, so it was difficult to observe the regular structure of MnO_2_.

Figure 3e,f shows the TEM analysis for the prepared MnO_2_/rGO-MWCNT/NF nanocomposite electrode. It indicated that the surface of the film showed 3D flowerlike structures staggered with each other (Supplementary Materials, Figure S3). The flowerlike hierarchical nanostructure maintains good integrity because the CNTs prevent rGO from restacking; therefore, the prepared MnO_2_/rGO-MWCNT/NF materials have more uniform dispersion and homogeneity [31]. Following examination by high-resolution TEM, the lattice patterns for rGO and MWCNT were observed, which showed the interplanar spacing for rGO and MWCNT to be approximately 0.40 nm and 0.35 nm, respectively. This result is very consistent with the XRD results discussed in the previous section.

**Figure 4:**
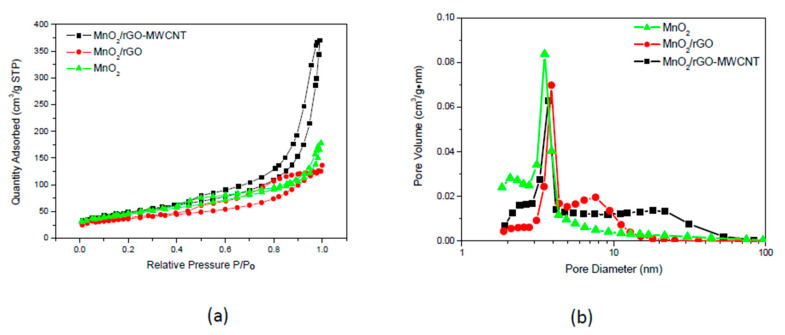
BET specific surface area and pore size distribution analysis for MnO_2_, MnO_2_/rGO and MnO_2_/rGO-MWCNT materials. (**a**) N_2_ isotherm adsorption-desorption analysis and (**b**) pore distribution analysis.

Furthermore, it was also found that the MWCNTs attach to the inside of MnO_2_, which indicates structural stability for the MnO_2_/rGO-MWCNT/NF nanocomposite electrode. Such an electrode structure can shorten the ion/electron transport length between the electrode and the electrolyte and increase the contact surface area. The 3D hierarchical flowerlike MnO_2_/rGO-MWCNT/NF nanocomposite electrode can be expected to show an excellent electrochemical performance. The energy dispersive spectrum (EDS) (Figure 3g) indicates that the MnO_2_/rGO-MWCNT material is composed of Mn, Ni, O and C, elements, except for Cu from the supporting Cu grid. It exhibits that the atomic composition of Mn and C is 15.4 at. % and 38.9 at. %, respectively; means that the loading amount of MnO_2_ in the MnO_2_/rGO-MWCNT material is approximately 74 wt. %. Furthermore, the Mn element in the electrode dispersed well in the hybrid material (Figure 3h,i), indicating that MnO_2_ has good dispersibility in the rGO-MWCNT. BET specific surface area and pore size distribution analysis for MnO_2_, MnO_2_/rGO and MnO_2_/rGO-MWCNT materials scraped from the as-deposited electrodes is shown in Figure 4. Figure 4a shows the N_2_ isotherm adsorption-desorption analysis for the MnO_2_-based materials. The results show a typical type IV isotherm. The sample has a clear triangular hysteresis loop and a steep absorption between 0.4–0.9 P/P_o_, showing highly interconnected holes, and the material has a narrow opening and a wide structure [32].

Table 1 shows the powder properties of MnO_2_, MnO_2_/rGO and MnO_2_/rGO-MWCNT materials. For the table, the specific surface area and pore size distribution were obtained by using the BET equation; the pore volume and the average pore diameter were obtained from the branch-curves for the adsorption isotherm according to the Barrett–Joyner–Halenda (BJH) equation. The results show that the BET specific surface areas for the MnO_2_, MnO_2_/MWCNT, MnO_2_/rGO, and MnO_2_/rGO-MWCNT materials were 155.7 m^2^·g^−1^, 102.6 m^2^·g^−1^, 132.8 m^2^·g^−1^, and 167.7 m^2^·g^−1^, respectively and that the BJH pore sizes were 4.6 nm, 5.6 nm, 6.7 nm, and 13.6 nm, respectively. MnO_2_/rGO-MWCNT showed the largest BET specific surface area, which is attributed to the loosely arranged structure; the MnO_2_/rGO specific surface area is small because graphene restacks.

In contrast, the MnO_2_/rGO exhibits the smallest specific surface area. It can be explained by combined with the results of Raman shifts (Supplementary Materials, Figure S1). It indicates that the relative intensity ratio of the D-band to G-band, defined as R = I_D_/I_G_, of the commercial GO sheet was found to be 0.86. This indicates that the GO showed the characteristic of high regularity and low randomness. Then, the as-obtained rGO by the hydrothermal method showed a highly stacked sp^2^ structure because of the elimination of the oxygen-containing functional groups, causing an increase in the reduction of graphite and a shift in the G band to 1585.5 cm^−1^.

Furthermore, the R-value of MnO_2_/rGO material increased after the SILAR process. That was presumably because some impurities and defects were introduced during the preparation. Moreover, the composited MnO_2_ particles also damaged the graphene order. Additionally, in the preparation of MnO_2_/rGO material, rGO was sequentially immersed in the MnSO_4_ and KMnO_4_ solutions by the SILAR process. Due to the high ionic strength of the solutions, the graphene was restacked and more agglomerated, resulting in specific surface area decrease.

Figure 4b shows the pore distribution analysis for MnO_2_, MnO_2_/rGO and MnO_2_/rGO-MWCNT materials. It can be seen from the figure that the pore distributions for the three different electrode materials were 2–8 nm, 5–15 nm and 2–75 nm, respectively. Due to the wide distribution of the MnO_2_/rGO-MWCNT material composition, it indicates the existence of a hierarchical porous structure. Especially for the MnO_2_/rGO-MWCNT material, the addition of MWCNT and rGO enhances the superb distribution of micropores, mesopores and macropores, and greatly improves the electrochemical performance.

The electrolyte ions’ size and the porous structure of the electrode are the key factors for the specific capacitance. The small size of the pores confines the accessible pores for the ions from the electrolyte solution. Su et al. [33] indicated that it is very important to meet the balance between the pore size and the ion size of the electrolyte for supercapacitors. However, an electrode material with hierarchical pore structure and high ion-accessible surface area that is helpful to ion transportation, and leading to high capacitance. It has been previously indicated that an ideal electrode material should have a hierarchical porous structure consisting of large pores (greater than 50 nm) used as an ion buffer reservoir, mesopores (2–50 nm) used for ionic migration and micropores (less than 2 nm) used for charge storage [34].

Xu et al. [35] also studied the supercapacitive properties of MnO_2_ electrode in Li_2_SO_4_, Na_2_SO_4_ and K_2_SO_4_ electrolyte, respectively. They indicated that the charging-discharging rate can be affected by the size of the cation, the size of the hydrated cation, the mobility of the cation, and the adsorption-desorption rate. The lithium ion (Li^+^) and hydrated lithium ion (Li_h_^+^) radius was 0.69 Å and 6 Å, respectively; sodium ion (Na^+^) and hydrated sodium ion (Na_h_^+^) radius was 1.02 Å and 4 Å, respectively, and potassium ion (K^+^) and hydrated potassium ion (K_h_^+^) radius was 1.38 Å and 3 Å, respectively. Therefore, Na^+^ ion may possess a moderate diffusion rate in MnO_2_ matrix, a moderate adsorption-desorption rate, and a moderate mobility in aqueous solutions, resulting in the large capacitance and fast charging/discharging rate.

Thus, in the as-obtained MnO_2_/rGO-MWCNT material, MWCNT helps prevent rGO from restacking and increases the specific surface area. The material has a highly hierarchical porous structure, which provides effective transportation for electrons and ions. MnO_2_/rGO-MWCNT/NF as an electrode in Na_2_SO_4_ electrolyte exhibits the higher specific capacitance as shown on Section 3.2 electrochemical properties.

### 3.2. Electrochemical Properties

**Figure 5:**
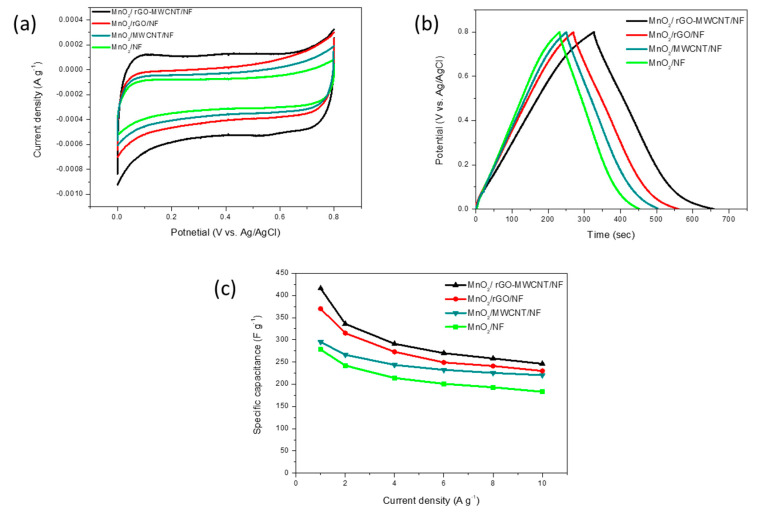
The capacitance performance of the as-deposited electrodes. (**a**) Cyclic voltammetry (CV) characteristics in a 1 M Na_2_SO_4_ electrolyte at a scan rate of 5 mV·s^−1^, (**b**) galvanostatic charging-discharging (GCD) characterization at 1 A·g^−1^; and (**c**) the relationship between the specific capacitance and current density from GCD examination.

These MnO_2_-based/NF materials were directly manufactured into working electrodes without using a binder to reveal their capacitance performance as shown in Figure 5. Figure 5a shows the analysis for the CV characteristics obtained for MnO_2_/NF, MnO_2_/MWCNT, MnO_2_/rGO/NF and MnO_2_/rGO-MWCNT/NF in a 1 M Na_2_SO_4_ electrolyte at a scan rate of 5 mV s^−1^, respectively.

The figure indicates the different electrodes with quasi-rectangular and quasi-symmetric CV curves. Among them, the area enclosed by the CV curve for the MnO_2_/rGO-MWCNT/NF electrode is much larger than the curve areas for the MnO_2_/NF, MnO_2_/MWCNT/NF and MnO_2_/rGO/NF nanocomposite materials, showing that the MnO_2_/rGO-MWCNT/NF electrode had an extremely high specific capacitance.

In the CV curve, MnO_2_/NF, MnO_2_/MWCNT/NF and MnO_2_/rGO/NF shows obvious gradient patterns in the voltage ranges of 0.0 V to 0.1 V and 0.7 to 0.8 V, individually, while the curve for the MnO_2_/rGO-MWCNT/NF can be observed to show an almost vertical CV characteristic curve with a near rectangular and symmetrical shape, indicating that the conductivity of MnO_2_/rGO-MWCNT/NF was greatly improve by the homogeneous mixing of rGO and MWCNT with MnO_2_, demonstrating excellent electrochemical performance. In addition, the CV curve areas for MnO_2_/rGO-MWCNT/NF are much larger than the CV curve areas for the MnO_2_/rGO/NF, MnO_2_/MWCNT/NF and MnO_2_/NF composites, respectively. It is speculated that both MnO_2_ and MWCNT can be used as spacers to prevent aggregation of rGO, which is conducive towards obtaining a higher electric double layer capacitance for rGO. Furthermore, rGO and MWCNT act as electronic conduction channels to increase the conductivity of MnO_2_. This is proved by the EIS analysis below, which shows a low contact electrical resistance for the MnO_2_/rGO-MWCNT/NF electrode.

Na_2_SO_4_ was used as electrolyte in this study, and the MnO_2_-based/NF electrodes showed a quasi-rectangular shape in CV curves measured at different scan rates, indicating the capacitance characteristics for the MnO_2_ deposited onto the NF electrode [36]. The CV curve for the MnO_2_-based electrode in Na_2_SO_4_ electrolyte is unlike that expected from an electric double-layer capacitor (EDLC); the CV characteristic curve for an EDLC shows a nearly ideal rectangle [37]. The quasirectangular shape observed for the CV curve is a characteristic of the reversible surface redox reaction of MnO_2_, the oxidation of Mn(III) to Mn(IV) and the reduction from Mn(IV) to Mn(III) [38].

Figure 5b shows GCD characterization for the as-obtained MnO_2_/NF, MnO_2_/MWCNT/NF, MnO_2_/rGO/NF and MnO_2_/rGO-MWCNT/NF electrodes at 1 A·g^−1^. The longer discharge time of the MnO_2_/rGO-MWCNT/NF electrode indicates that its capacitance was higher than that of the MnO_2_/NF, MnO_2_/MWCNT/NF and MnO_2_/rGO/NF electrodes, which is consistent with the results obtained from CV tests. In particular, compared with the as-prepared electrodes, the MnO_2_/rGO-MWCNT/NF electrode exhibits highly linear and almost symmetrical charge and discharge curves, revealing that the IR potential drop for MnO_2_/rGO-MWCNT/NF is less noticeable. The as-obtained MnO_2_/rGO-MWCNT/NF electrode has a maximum specific capacitance of 416 F·g^−1^ at a low current density of 1 A·g^−1^. Figure 5c further compares the relationship between the specific capacitance and current density determined from GCD examination. It can be found that, as the current density increases, the capacitance retention for the MnO_2_/rGO-MWCNT/NF electrode was better than that for the MnO_2_/NF, MnO_2_/MWCNT/NF and MnO_2_/rGO/NF electrodes. At a high current density of 10 F·g^−1^, the specific capacitance of the MnO_2_/rGO-MWCNT/NF electrode remained at 250 F·g^−1^, while the MnO_2_/NF, MnO_2_/MWCNT/NF and MnO_2_/rGO/NF electrodes showed a capacitance of 176 F·g^−1^, 215 F·g^−1^ and 232 F·g^−1^, respectively.

There was no oxidation peak/reduction peak in the CV curves and the GCD discharge curve of 0.0–0.2 V indicates a similar characterization of slope variation of the time for the as-deposited MnO_2_-based electrode as Na_2_SO_4_ used for electrolyte (Figure 5a,b). This is due to the charge transfer reaction between MnO_2_ and Na_2_SO_4_ electrolyte, which is related to the pseudo-capacitance behavior [39].

Contrast to the MnO_2_ electrode in KOH electrolyte, the oxidation peak/reduction peak appears in the CV curve, and the steep slope at the end of the GCD discharge curve [40]. It reported that redox reaction peaks were visible (in the CV curves), indicating that the process of energy storage was mainly associated with a pseudocapacitance mechanism and not the reaction between the Mn^4+^ and OH^−^ in the electrolyte [41]. The redox mechanism of MnO_2_ in KOH electrolyte are reversible insertion/extraction of K^+^ in MnO_2_ as Formula (3) [42]:

MnO_2_ + K^+^ + e^−^ ↔ MnOOK (3)

**Figure 6:**
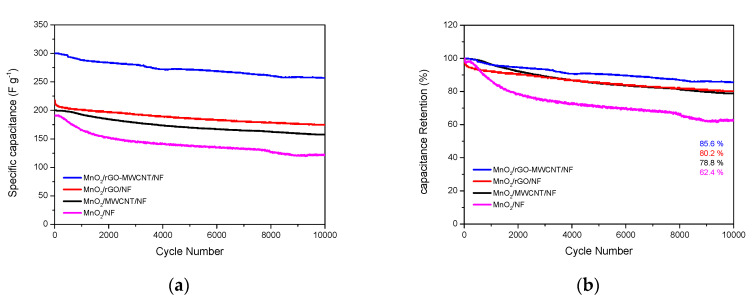
The cycling charge-discharge test for the as-deposited electrodes in a 1 M Na_2_SO_4_ electrolyte at a constant current of 4 A·g^−1^. (**a**) Specific capacitance change and (**b**) capacitance retention.

Figure 6 shows the cycling charge-discharge test for the MnO_2_/NF, MnO_2_/rGO/NF and MnO_2_/rGO-MWCNT/NF electrodes in a 1 M Na_2_SO_4_ electrolyte at a constant current of 4 A·g^−1^. The results clearly show that the specific capacitance of the MnO_2_/rGO-MWCNT/NF electrode decreased at 10,000 cycles of charging and discharging; however, this decrease was smaller than that found for the MnO_2_/rGO/NF and MnO_2_/NF electrodes, respectively. The MnO_2_/NF, MnO_2_/MWCNT/NF, MnO_2_/rGO/NF and MnO_2_/rGO-MWCNT/NF electrodes exhibit a capacitance retention of 62.4% (from 194 to 121 F·g^−1^), 78.8% (from 201 to 158 F·g^−1^), 80.2% (from 223 to 179 F·g^−1^), and 85.6% (from 302 to 259 F·g^−1^), respectively.

In this study, SILAR technology was used to prepare a MnO_2_/rGO-MWCNT/NF electrode onto rGO-MWCNT composite coated foamed nickel substrates. The material was found to show excellent cycle stability, which verifies that the charge storage reaction of the supercapacitor is reversible and that the electroactive material is stably adsorbed onto the substrate (current collector). MnO_2_/rGO-MWCNT/NF can maintain high cyclic stability, which can be mainly attributed to the synergy effect between rGO, MWCNT and MnO_2_.

Kong et al. [43] indicated that in the use of graphene nanosheets (GNS) to produce electrode materials, the aggregation and restacking of GNS will hinder the migration of electrolyte ions onto the interface, resulting in a substantial decrease in electrochemical performance. When multiwall carbon nanotubes (MWCNTs) are introduced, the graphene layer can be dispersed and the diffusion coefficient for the ions in the material can be effectively improved. In addition, the synergy effect between GNS and MWCNT is conducive to increasing the contact area between the electrode material and the electrolyte, providing a rich electroactive site for pseudocapacitance; such a hierarchical porous structure can effectively shorten the Na^+^ diffusion path. Sun et al. [44] studied a MnO_2_/rGO/Ni composite foam electrode exhibiting good supercapacitor performance. It was pointed out that this excellent performance was closely related to the inherent hierarchical nanostructured porous MnO_2_/rGO composite material grown onto the foamed Ni framework.

In this study, the SILAR process was used to apply layer-by-layer coating technology to prepare MnO_2_/rGO-MWCNT/NF electrodes. In addition to the aforementioned characteristics [43,44], SILAR is more capable of producing a high surface area material with a higher electrolyte diffusion rate; in addition, rGO-MnO_2_ electrodes prepared using SILAR technology can be used to manufacture lightweight and ultrasmall supercapacitor devices. It can induce the material to be more uniformly dispersed, increasing the capacity to build MnO_2_/rGO-MWCNT/NF electrodes that demonstrate excellent cycle durability and excellent electrochemical performance.

**Figure 7:**
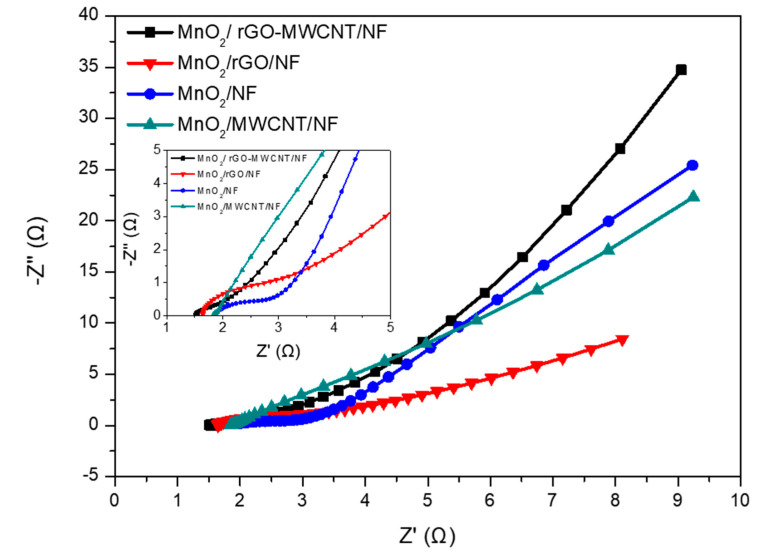
Electrochemical impedance spectra (EIS) analysis of the as-deposited electrodes in the frequency range from 100 kHz to 0.01 Hz.

To study the electrochemical mechanism for the MnO_2_-based composite electrode materials showing good supercapacitor properties, MnO_2_/NF, MnO_2_/MWCNT/NF, MnO_2_/rGO/NF and MnO_2_/rGO-CNT/NF electrodes were prepared and subjected to EIS analysis, as shown in Figure 7. EIS measurements were taken in the frequency range from 100 kHz to 0.01 Hz. The results were displayed using Nyquist plots, which are divided into three different regions:

In the high frequency region, the intercept at the real axis (Z_0_) represents the equivalent series resistance (ESR), including the ionic resistance of the electrolyte, the inherent resistance of the substrate, and the contact resistance of the active material/current collector interface [45]. The span of the semicircular arc in the mid-high frequency region represents the charge transfer resistance (R_ct_) at the electrode/electrolyte interface, also known as the Faraday resistance [46,47]. In the low-frequency region, the impedance represents the diffusion resistance for the electrolyte ions in the holes of the electrode. If the impedance graph increases sharply and tends to become a vertical line, a characteristic of pure capacitance behavior is indicated [48].

As shown in Figure 7, at high frequencies, the intercepts (R_E_) for the curve and real axis for the MnO_2_/rGO-MWCNT/NF composite electrode, MnO_2_/rGO/NF, MnO_2_/MWCNT/NF and MnO_2_/Ni electrodes were determined to be 1.5 Ω, 1.7 Ω, 1.9 Ω and 2.1 Ω, representing a good contact between the electrode and the electrolyte, respectively. Especially, the MnO_2_/rGO-MWCNT/NF composite electrode shows the smallest equivalent resistance, demonstrating that the electrode has better conductivity. In particular, the as-obtained MnO_2_/rGO-MWCNT/NF composite electrode shows a vanishing semicircular arc-shaped impedance in the high-medium frequency region, indicating that the charge transfer resistance (R_ct_) for the electrode is extremely low and that the ion diffusion path is very short. This result has hardly been observed previously in the high-to-medium frequency region, which is similar to the study of Liu et al. [49].

In the low-frequency region, the MnO_2_/rGO-MWCNT/NF electrode shows a straight line with a steep slope, indicating that the capacitance performance is very close to that of an ideal supercapacitor [50]. Additionally, in this region, the slope of the impedance curve for the MnO_2_/rGO/NF electrode is not as steep as that for the other electrodes, which may be due to the relatively worse dispersion of the rGO in the MnO_2_/rGO/NF electrode.

In this study, the as-obtained MnO_2_/rGO-MWCNT/NF electrode exhibited an extremely low impedance, which is attributed to the high homogeneity and nanostructure of the hierarchical porous composites grown on the nickel foam. The addition of MWCNTs leads to high aggregation but high specific surface area rGO is easily dispersed, unclogging the electronic conductive channels, resulting in an extremely small (even difficult to observe) arc span for the MnO_2_/rGO-MWCNT/NF electrode. In addition, the porous hierarchical structural MnO_2_/rGO-MWCNT/NF electrode exhibits an equivalent series resistance (R_E_) that is lower than that of the other electrodes. This result further shows that the composite MnO_2_/rGO-MWCNT/NF electrode has faster kinetics compared to the MnO_2_/rGO/NF, MnO_2_/MWCNT/NF and MnO_2_/NF composite electrode, which is beneficial towards improving the capacitance performance of the composite material, especially at high charge/discharge rates for the supercapacitor [51,52].

In addition, comparing the electrochemical properties of MnO_2_/MWCNT/NF and MnO_2_/rGO/NF electrodes, it is found that MnO_2_/rGO/NF exhibits relatively good specific capacitance; however, the capacitance retention of MnO_2_/MWCNT/NF electrode seems to be relatively stable. It is postulated that in the MnO_2_/rGO/NF electrode, the MnO_2_ nanoparticles make the rGO exhibit relatively good exfoliations, which makes the MnO_2_ deposition relatively dispersed, and the material has a higher specific surface area. The electrode possesses a better pore structure makes Na_2_SO_4_ electrolyte easily adsorbed on the electrode, which facilitates migration and diffusion; resulting in a larger CV curve area and a higher specific capacitance value. In contrast, in MnO_2_/MWCNT/NF, it is possible that MnO_2_ is relatively easy to firmly adhere to the wall of the MWCNT, and the electrode microstructure is relatively strong, therefore, the capacitance retention during the charge-discharge cycling is relatively stable. However, the detailed differences between MnO_2_/MWCNT/NF and MnO_2_/rGO/NF need to be studied more accurately.

Table 2 shows a comparison of electrochemical performance of MnO_2_-based electrode in the literatures [6,53,54,55,56]. Galvanostatic charge-discharge measurement results revealed that the composite with hybrid MnO_2_/rGO-CNT exhibited the specific capacitance of 416 F·g^−1^ at 1 A·g^−1^ in a 1 M Na_2_SO_4_ electrolyte and an excellent capacitance retention of 85.6% at 10,000 charge-discharge cycles. The capacitance retention is quite higher than that of the previously studied electrode material.

Combining the above results, the hybrid nanostructural rGO-MWCNT and MnO_2_ material on nickel foam enables fast electron and ion transportation and further improves electrochemical performance. The addition of MWCNTs leads to high aggregation but also to the easy dispersion of rGO. The 3D network structure of the MnO_2_/rGO-MWCNT/NF electrode was found to exhibit an excellent pore distribution, which can facilitate passage of electrons, charge storage and electron transportation. Therefore, MnO_2_/rGO-MWCNT/NF composites were successfully synthesized, which display enhanced electrochemical performance as potential electrode materials for supercapacitors.

## 4. Conclusions

SILAR technology was used to construct 3D δ-MnO_2_-based/foamed nickel electrodes. A hierarchical porous MnO_2_ nanocomposite electrode material was supported on rGO-coated and rGO-MWCNT-coated nickel foam by convenient and simple “immersion and drying”, respectively. Because MWCNTs can effectively enable rGO to form a stable dispersion, they are beneficial for the uniform deposition of MnO_2_ onto the substrate.

The synergetic combination of rGO-MWCNT and pseudocapacitance MnO_2_ material onto nickel foam enables fast electron and ion transportation and further improves electrochemical performance. The as-prepared MnO_2_/rGO-MWCNT/NF electrode was found to exhibit extremely low impedance due to the high uniformity and nanostructured material properties of the porous composite material grown onto the nickel foam. The addition of MWCNTs leads to high aggregation but also to the easy dispersion of high specific surface area rGO. The 3D network structure of the MnO_2_/rGO-MWCNT/NF electrode was found to exhibit an excellent pore distribution, which can facilitate passage of electrons, charge storage and electron transportation.

The as-deposited MnO_2_/rGO-MWCNT/NF hierarchical porous nanostructural electrode exhibited a high specific capacitance of 416 F·g^−1^ at 1 A·g^−1^ in 1 M Na_2_SO_4_. After 10,000 charge and discharge cycles, the capacitance retention reached 85.6%. Therefore, this high-performance and convenient fabrication method provides excellent prospects for energy storage applications.

## Figures and Tables

**Table 1:** The powder properties of MnO_2_, MnO_2_/MWCNT, MnO_2_/rGO and MnO_2_/rGO-MWCNT materials.

Materials	Surface Area (m^2^·g^−1^)	Pore Volume (cm^3^·g^−1^)	Pore Size (nm)
MnO_2_	155.7	0.4	4.6
MnO_2_/MWCNT	102.6	0.3	5.6
MnO_2_/rGO	132.8	0.2	6.7
MnO_2_/rGO-MWCNT	167.7	0.5	13.6

**Table 2:** Comparison of electrochemical performance of MnO_2_-based electrode in the literatures.

Material	Electrolyte	Current Density (A/g)	Capacitance Retention (%)	Specific Capacitance (F/g)	Ref.
MnO_2_	0.5 M Na_2_SO_4_	1		336	[53]
MnO_2_/rGO	1 M Na_2_SO_4_	0.5	94.7 (1000 cycles)	288	[6]
MnO_2_/rEGO	1 M Na_2_SO_4_	0.5	90.3 (1000 cycles)	99.5	[54]
δ-MnO_2_/GO	1 M Na_2_SO_4_	1	81 (1000 cycles)	255	[55]
MnO_2_/CNTs	1 M Na_2_SO_4_	0.2	90 (2000 cycles)	162	[56]
MnO_2_/rGO-CNT	1 M Na_2_SO_4_	1	85.6 (10,000 cycles)	416	This work

rEGO: reduced graphene oxide obtained from electrochemical exfoliation method.

## Supplementary Materials

The following are available online at https://www.mdpi.com/2079-4991/10/10/1933/s1, Figure S1: Properties of the as-obtained materials; Figure S2: Material properties and electrochemical characterization of the as-obtained MnO_2_ from different concentrations of MnSO_4_; Figure S3: FESEM images for the as-obtained MnO_2_-based/NF materials.
