# Supplementary material for: Enhanced Activity of Hierarchical Nanostructural Birnessite-MnO2-Based Materials Deposited onto Nickel Foam for Efficient Supercapacitor Electrodes
Source: Nanomaterials (Basel). 2020 Sep 27;10(10):1933. doi: 10.3390/nano10101933 (PMC7599501; doi:10.3390/nano10101933)
Supplement: Supplementary file 1 [file nanomaterials-10-01933-s001.pdf]

Supplementary Materials

Enhanced Activity of Hierarchical Nanostructural Birnessite-MnO_2-Based Materials Deposited onto Nickel Foam for Efficient Supercapacitor Electrodes

Shang-Chao Hung [1], Yi-Rong Chou [2], Cheng-Di Dong [3], Kuang-Chung Tsai [4] and Wein-Duo Yang [2,*]

[1] Intelligent Technology Research Centre, Fuzhou Polytechnic, Fuzhou 350108, China; schung99@gmail.com
[2] Department of Chemical and Materials Engineering, National Kaohsiung University of Science and Technology, Kaohsiung 80778, Taiwan; mygirl850629@gmail.com
[3] Department of Marine Environmental Engineering, National Kaohsiung University of Science and Technology, Kaohsiung 81157, Taiwan; cddong@nkust.edu.tw
[4] Department of Safety, Health and Environmental Engineering, National Kaohsiung University of Science and Technology, Kaohsiung 82445, Taiwan; tsaikc@nkust.edu.tw
* Correspondence: ywd@nkust.edu.tw; Tel.: +886-7-3814526 (ext. 15116)

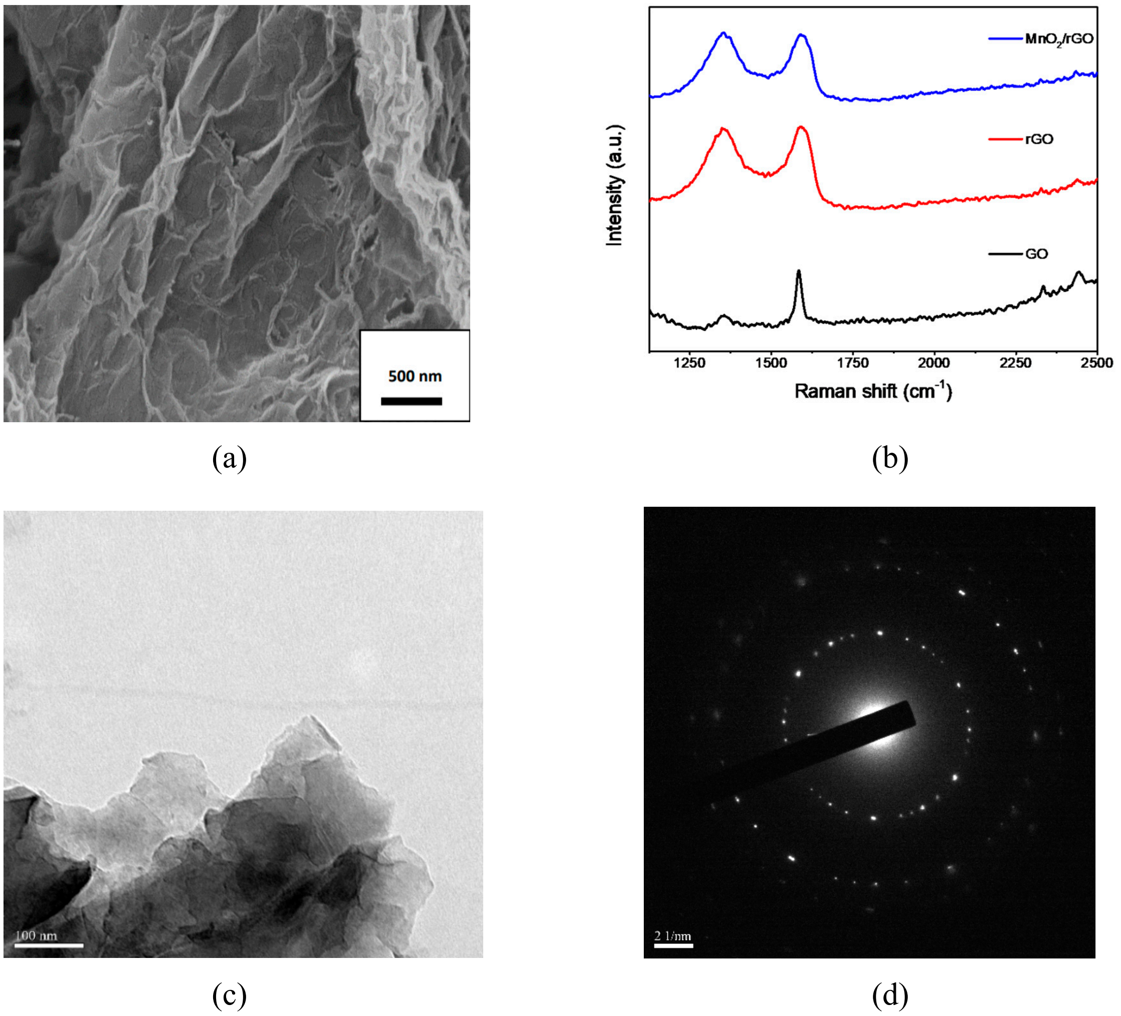

Figure S1. The properties of the as-obtained materials. (**a**) HRSEM image of the as-prepared rGO; (**b**) Raman shift of GO (R = 0.86, G band at 1597.8 cm^{-1}), rGO (R = 0.87, G band at 1585.5 cm^{-1}, and MnO_2/rGO (R = 1.02, G band at 1587.3 cm^{-1}; (**c**) TEM image of as-prepared rGO; and (**d**) electron diffraction patterns of rGO.

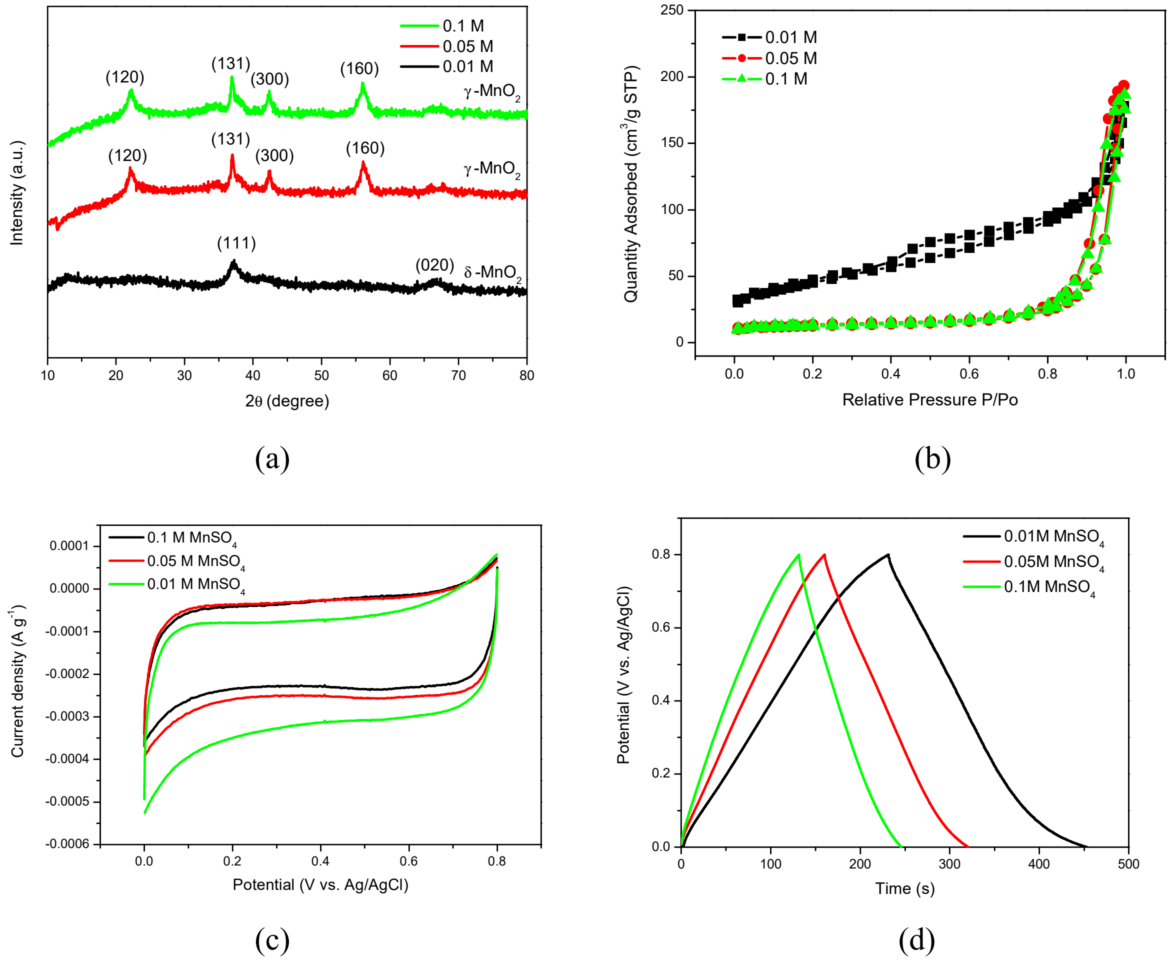

Figure S2. The material properties and electrochemical characterization of the as-obtained MnO_2 from different concentrations of $MnSO_4$. (**a**) XRD analysis, (**b**) BET N_2 absorption-desorption analysis, (**c**) C-V curve characterization of MnO_2, and (**d**) GCD test.

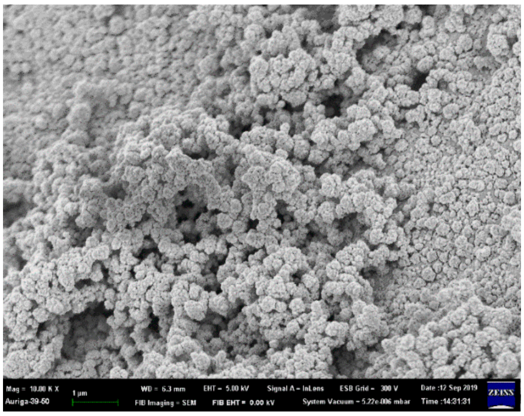

(a)

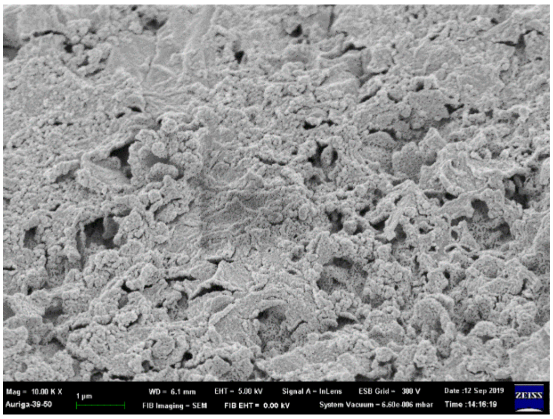

(b)

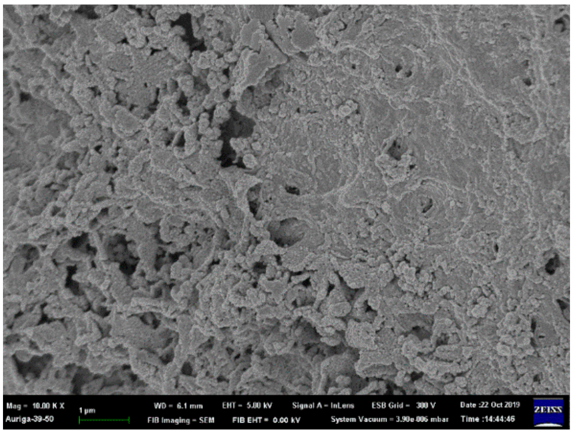

(c)

Figure S3. The FESEM images for the as-obtained MnO_2/NF material (**a**), MnO_2/rGO/NF material (**b**) and MnO_2/rGO-MWCNT/NF electrode (**c**), respectively.
